# Introduction

**DOI:** 10.1080/09629359791541

**Published:** 1997-11

**Authors:** Hajime Goto, Ragnar Rylander


Mediators of Inflammation, **6,** 245 (1997)

### Introduction

**Hajime Goto and Ragnar Rylander**

The proceedings here are from the fourth workshop on the toxicity of (1→3)-β-D-glucan organized by the Committee on Organic Dusts at the International Commission on Occupational Health. Previous workshops have established a basis for the understanding how (1→3)-β-D-glucan can interfere with cells and how pulmonary pathology can arise [1–3]. Field studies have also been an important data resource as have clinical investigations on the therapeutic effects of (1→3)-β-D-glucan.

There is an unusual enthusiasm among several researchers for this 'new' agent in the occupational and general environment and for how it could explain symptoms and disease. Previous experience demonstrates, however, that there are many pitfalls along the way and that important questions regarding sampling and species differences between different kinds of (1→3)-β-D-glucan need to be clarified.

It is hoped that these proceedings clarify some of these basic problems in addition to presenting new data from ongoing experimental and epidemiological research. We are especially grateful to the participants who worked hard for 2 days at this workshop.

### References

1. Rylander R, Goto H (eds): *First Glucan Lung Toxicity Workshop. Committee on Organic Dusts ICOH, Report 1/91*. Gothenburg, Sweden: Department of Environmental Medicine, University of Gothenburg; 1991.
2. Rylander R, Peterson Y (eds): *Second Glucan Inhalation Toxicity Workshop. Committee on Organic Dusts ICOH, Report 1/93*. Gothenburg, Sweden: Department of Environmental Medicine, University of Gothenburg; 1993.
3. Rylander R, Goto H (eds): *Third Glucan Inhalation Toxicity Workshop. Committee on Organic Dusts ICOH, Report 1/94*. Gothenburg, Sweden: Department of Environmental Medicine, University of Gothenburg; 1994.

© 1997 Rapid Science Publishers

